# Automated 5-plex fluorescent immunohistochemistry with tyramide signal amplification using antibodies from the same species

**DOI:** 10.1186/2051-1426-3-S2-P111

**Published:** 2015-11-04

**Authors:** Wenjun Zhang, Antony Hubbard, Tobin Jones, Srabani Bhaumik, Adriana Racolta, Mark Lefever, Karl Garsha, Nicholas Cummins, Franklin Ventura, Lei Tang

**Affiliations:** 1 Roche Diagnostics, Tucson, AZ, USA

## Background

Immunohistochemical (IHC) detection of multiple antigens on the same tissue section represents a major unmet technological need in research and clinical diagnostics. While primary antibodies from different species have been used with differently labeled species-specific secondary antibodies, quite often the appropriate combination of antibodies is not available. More recently, primary antibodies from the same species have been employed for multiplex IHC with microwaving treatment between each antigen staining cycle. This manual microwaving method prevents the cross-reactivity of an anti-species antibody in a subsequent cycle from binding to a primary antibody at the previous cycle.

## Methods

We present here a fully automated heat deactivation (HD) process on the BenchMark ULTRA automated slide stainer. We verified various aspects of the HD process: (1) effectiveness for preventing cross-reactivity, (2) impact on the epitopes in tissues, (3) impact on the fluorescence of the fluorophores, and (4) impact on tissue morphology. We further validated an automated 5-plex fluorescent IHC assay for CD3, CD8, CD20, CD68 and FoxP3 on human tonsil tissues.

## Results

This automated 5-plex fluorescent IHC assay using rabbit primary antibodies achieved comparable staining results to the respective single-plex chromogenic IHC assays. This technology enables a simple automated multiplex IHC assay through the use of commercially available native primary antibodies with their respective secondary anti-species antibody, and a clinically proven staining platform to ensure staining quality, reliability, and reproducibility.

